# Neuron‐derived small extracellular vesicles as a ‘liquid biopsy’ to assess brain insulin signaling deregulation in Alzheimer's disease pathogenesis

**DOI:** 10.1002/alz.091922

**Published:** 2025-01-09

**Authors:** Jacob Alexander Cleary, Ashish Kumar, Yixin Su, Susy Kim, Sangeeta Singh, William Harrison, Kathleen M. Hayden, Jingyun Lee, Mark A. Espeland, Cristina M Furdui, Jingzhong Ding, Suzanne Craft, Gagan Deep

**Affiliations:** ^1^ Wake Forest University School of Medicine, Winston Salem, NC USA; ^2^ Wake Forest University School of Medicine, Winston‐Salem, NC USA

## Abstract

**Background:**

Insulin signaling deregulation in the brain is a critical risk factor for Alzheimer's disease (AD); however, molecular changes in this pathway during AD pathogenesis cannot be currently accessed in clinical setting due to lack of brain tissues. Here, we propose small extracellular vesicles (sEV) characterization as a non‐invasive approach to assess the status of insulin signaling in the AD brain.

**Method:**

In postmortem brain tissues of cognitively normal (CN) and AD (n=5 each) subjects, expression of 84 genes, involved in insulin signaling and resistance was analyzed using pathway specific PCR array. Next, the level of key miRNAs (miR185‐5p, miR‐210‐3p, and miR342‐3p), that regulate the expression of insulin signaling genes, was assessed by TaqMan‐based qPCR in brain tissues and secreted sEV in their microenvironment. The expression of these miRNA was also analyzed in the neuron‐derived sEV (NDE) isolated from the plasma of 28 CN (18 female and 10 male) and 28 dementia (11 female and 17 male) subjects, all with type‐2 diabetes. These highly specific NDE were isolated by sequential immunoprecipitation using CD171 and synaptophysin surface markers, and the expression of studied miRNAs was correlated with corresponding clinical measures (MMSE score, Aβ1‐40 and Aβ1‐42 levels).

**Result:**

AD brain tissue revealed significant deregulation of genes involved in insulin signaling and resistance (e.g., AKT2, GSK3β, INSR, KRAS, PIK3R1, IGF1R, PTPN1, BRAF, GCK, UCP1, LDLR, NPY, and RRAS2). We also observed a significant increase in the expression of miRNAs (miR185‐5p, miR‐210‐3p, and miR342‐3p) involved in regulation of these genes. Interestingly, the expression of these miRNAs in the brain tissues as well as their secreted sEV showed a high degree of concordance. Most importantly, NDE in the plasma of individuals with dementia also showed a similar change in the expression of miRNAs regulating insulin signaling in the brain, suggesting suitability of sEV application as liquid biopsy for the brain tissue. Lastly, this increased expression of NDE miR‐185‐5p in dementia showed significant inverse correlation with MMSE Score.

**Conclusion:**

NDE in plasma offer critical molecular information about insulin signaling and resistance in the brain, which could be valuable in making diagnostic and treatment decisions in AD.

